# Glycopolymer Brushes by Reversible Deactivation Radical Polymerization: Preparation, Applications, and Future Challenges

**DOI:** 10.3390/polym12061268

**Published:** 2020-06-01

**Authors:** Jessica P. M. Ribeiro, Patrícia V. Mendonça, Jorge F. J. Coelho, Krzysztof Matyjaszewski, Arménio C. Serra

**Affiliations:** 1 Department of Chemical Engineering, Centre for Mechanical Engineering, Materials and Processes, University of Coimbra, Rua Sílvio Lima-Polo II, 3030-790 Coimbra, Portugal; jessicamribeiro1@hotmail.com (J.P.M.R.); jcoelho3@gmail.com (J.F.J.C.); 2 Department of Materials Science & Engineering, Carnegie Mellon University, 5000 Forbes Avenue, Pittsburgh, PA 15213, USA; km3b@andrew.cmu.edu

**Keywords:** glycopolymer, brush-like polymers, reversible deactivation radical polymerization, lectin binding

## Abstract

The cellular surface contains specific proteins, also known as lectins, that are carbohydrates receptors involved in different biological events, such as cell–cell adhesion, cell recognition and cell differentiation. The synthesis of well-defined polymers containing carbohydrate units, known as glycopolymers, by reversible deactivation radical polymerization (RDRP) methods allows the development of tailor-made materials with high affinity for lectins because of their multivalent interaction. These polymers are promising candidates for the biomedical field, namely as novel diagnostic disease markers, biosensors, or carriers for tumor-targeted therapy. Although linear glycopolymers are extensively studied for lectin recognition, branched glycopolymeric structures, such as polymer brushes can establish stronger interactions with lectins. This specific glycopolymer topology can be synthesized in a bottlebrush form or grafted to/from surfaces by using RDRP methods, allowing a precise control over molecular weight, grafting density, and brush thickness. Here, the preparation and application of glycopolymer brushes is critically discussed and future research directions on this topic are suggested.

## 1. Introduction

**Figure 1:**
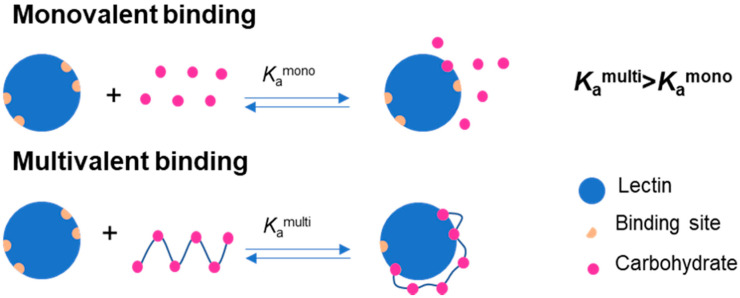
Schematic representation of the binding between lectins and one carbohydrate (monovalent binding) or two or more carbohydrates (multivalent binding).

The cellular surface is covered with different receptors, that mediate several recognition events in living systems through carbohydrate–protein, protein–protein, or antigen–antibody interactions [1]. Particular interest is devoted to carbohydrate-recognition proteins, also termed lectins, that can recognize carbohydrates (e.g., present at the cell surface) and participate in different biological events, namely cell–cell adhesion, cell–cell recognition and cell differentiation. Lectins represent the most investigated molecular target in the drug discovery field [2,3]. These structures were first termed in 1980 as carbohydrate-binding proteins of non-immune origin, which agglutinate cells or precipitate polysaccharides or glycoconjugates [4,5]. These macromolecules are multivalent proteins containing two or more carbohydrate-binding sites, which have been extensively investigated for their biological interaction with glyconanostructures, such as glycopolymers or glycopeptids [3,5,6]. Concanavalin A (Con A) is the most studied plant-based lectin and it can be obtained from jack beans. Its carbohydrate-binding specificity has been characterized in detail [4,5]. Con A is a mitogenic lectin because it can activate and induce the mitoses of T lymphocytes, which are important molecules for the assessment of immunocompetence of patients with immunodeficiency diseases [7]. The α-anomer of d-mannose is the most complementary monosaccharide to the Con A sugar-binding site. However, this lectin can also bind to d-glucose, d-fructose, *N*-acetyl-d-glucosamine, and related monosaccharides [4,5,8]. It is also known that the interactions are favored by monosaccharides in their pyranose form and with free hydroxyl groups at C-3, C-4, and C-6 positions [5,8]. The monovalent binding between lectins and one sugar residue is typically weak. However, lectins that possess two or more domains able to recognize carbohydrates can form multivalent binding that enhances the strength of interaction, which is known as the multivalent effect (Figure 1) [3,5,6,9].

**Figure 2:**
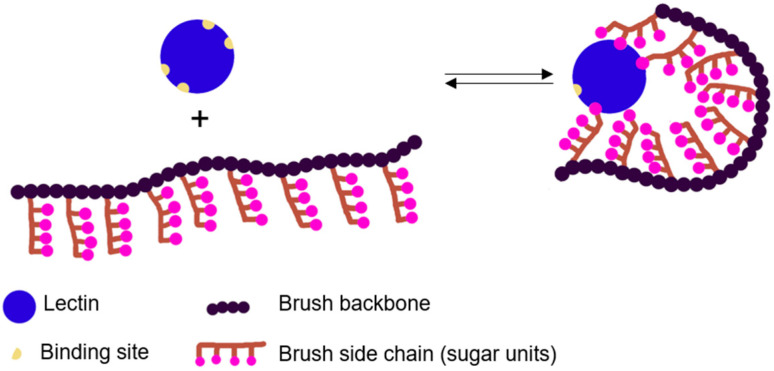
Schematic representation of the binding between a lectin and a glycopolymer brush chain by the multivalent effect.

Taking advantage of the lectin-carbohydrate recognition ability combined with the multivalent effect led to the development of synthetic polymeric structures with many pendant sugar groups, known as glycopolymers. These structures are promising candidates for the biomedical field, namely as novel drug delivery systems, by targeting lectins present on the cell surface [9,10]. These polymers can be prepared by either the direct polymerization of vinyl glycomonomers or by post-modification of functional polymers with carbohydrates-containing compounds [9,11,12]. Regarding the direct polymerization of vinyl glycomonomers, it can be carried out using monomers either in their protected or unprotected form, which are obtained through the functionalization of sugar molecules with reactive double bonds [12]. As carbohydrates have numerous functional groups, their protection and later deprotection requires multi-step reactions. The use of unprotected functional monomers allows the preparation of the polymer in a one-step process and avoids potential degradation during deprotection steps, that could influence the performance of the synthesized material [9,13]. Although there are reports on the polymerization of glycomonomers by free radical polymerization (FRP), reversible deactivation radical polymerization (RDRP) methods are the most often used for this purpose. Indeed, these advanced polymerization methods allow a fine control over the glycopolymer molecular weight, architecture, composition, and structure, which are known to influence their bioactivity because of the precise structure of the targeting proteins [12,14,15]. The most commonly used RDRP methods are atom transfer radical polymerization (ATRP), nitroxide-mediated polymerization (NMP), and reversible addition-fragmentation chain-transfer polymerization (RAFT) [9,12]. Nevertheless, most of the reports on the synthesis of glyco(co)polymers with different architectures, namely linear, star-shaped, or bottle brush are conducted by ATRP. Regarding polymer architecture, the most studied glycopolymeric structures are the linear ones [9]. However, their application in fields that require high recognition ability (e.g., biosensing or drug delivery) is actually limited compared to that of other polymer architectures, such as bottlebrushes. The latter provide higher surface area for the binding between the glycopolymer and lectins, (Figure 2) [10]. In fact, the bottlebrush architecture gives polymeric materials with unique properties: (i) high concentration of polymeric side chains (sugar units); (ii) stretched polymer backbone because of the steric repulsion between side chains; and (iii) decreased spatial density of chain entanglement [16]. These properties have opened exciting opportunities for the use of polymer brushes in applications, for which linear polymers were less effective, such as drug carriers, super-soft elastomers, surfactants, lubricants, and stimuli-responsive materials [16]. Glycopolymer-coated surfaces are especially relevant considering the development of materials with anti-adhesive properties for applications on the biomedical field (e.g., implants) [17]. Studies showed that regardless the methodology used for the synthesis of glycopolymer brushes, it is known that a high brush density is required to enhance lectin-binding, thus preventing non-specific protein, cellular and bacterial adhesion to coated surfaces [18].

This review presents and discusses the literature describing the preparation and application of well-defined brush-like glycopolymers using mono or disaccharides monomer structures, by RDRP techniques. First, a brief description of the RDRP strategies available for the synthesis of such glycopolymer architectures is provided. Then, the article is divided in different sections, according to the type of sugar used in the glycopolymer brush and within each section, the potential application of the materials is described, highlighting the importance of these materials, especially in the biomedical field.

## 2. Preparation of Polymer Brushes by RDRP

**Figure 3:**
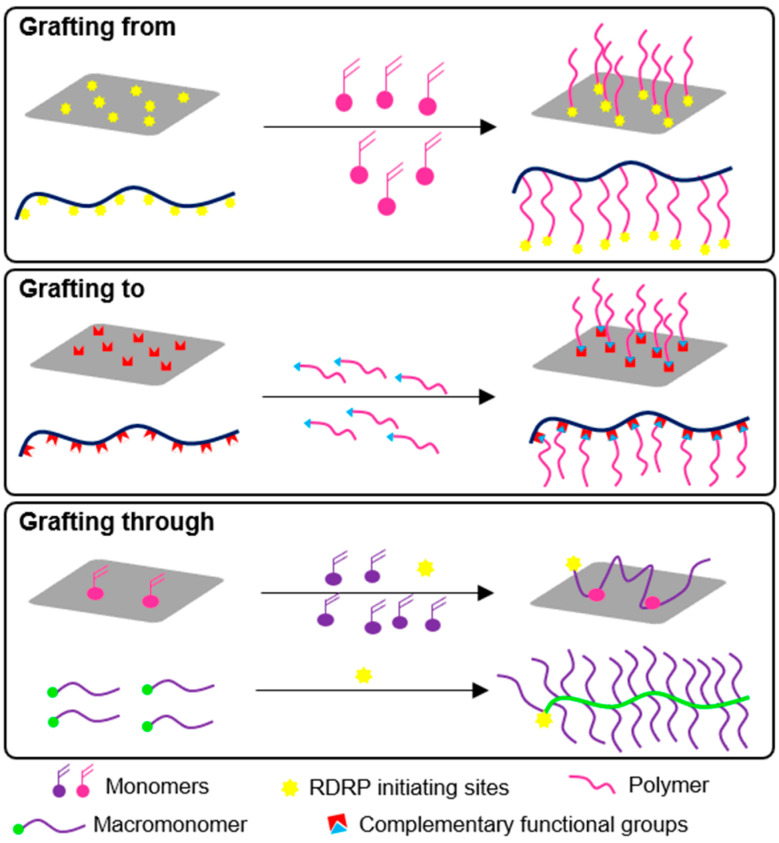
Available strategies for the preparation of polymeric bottlebrushes or immobilized polymer brushes by RDRP techniques: “grafting from”, “grafting to”, and “grafting through”.

Polymer brushes are densely grafted polymers that can be found attached to several solid surfaces [19] or in their free form, also known as bottlebrushes [20,21]. These polymers can be prepared using RDRP methods mainly by three strategies, “grafting to”, “grafting from”, and “grafting through”, (Figure 3) with extreme precision over molecular weight, grafting density, and grafting thickness [16,22]. In the “grafting to” approach, the polymeric side chains are synthesized separately by appropriate RDRP techniques, with functional chain-end groups complementary to the ones present on the polymer backbone or surface. In a second step, both functional polymeric side chains and backbone or surface will be covalently linked (e.g., using “click” chemistry methods), to form the polymer brush [16,22]. With “grafting to”, it is possible to characterize individually the backbone and side chains, but steric hindrance problems and slow diffusion of these chains associated with this strategy could lead to low grafting density, thus requiring additional purification steps to remove unreacted side chains. In the “grafting from” method, a monomer is polymerized directly from a surface or a polymer backbone with predefined initiation sites, from which the polymeric side chains will grow [16]. Usually, polymerizations are conducted under diluted conditions to ensure a low concentration of radicals, as well as small polymerization rate, in order to minimize possible termination reactions, which would lead to significant structural defects considering the large size of polymer brushes. Within the three approaches, the “grafting-from” is the preferred one for the synthesis of brushes with high grafting density. Finally, the “grafting through” consists on the polymerization of macromonomers to obtain bottlebrushes or the use of surfaces containing immobilized monomers for the incorporation of a growing polymer chain in solution, which can be linked to the surface through one or more points, depending on the degree of surface functionalization. While this could be a good strategy to modify some surfaces (e.g., glass), the “grafting through” seems to be less suitable when using, for instances, a dispersion of nanoparticles (NPs) immobilized with monomers, as the growing polymer can easily incorporate monomers from different NPs, thus affecting the homogeneity of the final brush. Interesting, there is also one work proposing a new “grafting through” method for the synthesis of polymers brushes based on monomer supply through porous surfaces [23]. There are few reports of the synthesis of glycopolymer brushes by grafting through, specially by RDRP methods. Detailed information on the preparation of polymer brushes immobilized in surfaces can be found in a review article [22].

The most common method employed for the preparation of glycopolymer brushes is the “grafting from” approach, mainly using surfaces functionalized with initiating sites that will determine the grafting density. Within RDRP techniques, surface-initiated (SI)-ATRP is the most often used method. ATRP and SI-ATRP have excellent tolerance to functional groups and can be performed with glycomonomers without any protection of the hydroxyl groups [10]. RAFT and SI-RAFT polymerizations can be also employed for the preparation of glycopolymeric structures, using a chain transfer agent with no need of a metal source in opposition to ATRP [10]. However, RAFT polymerization usually involves higher polymerization temperature than the one typically employed for ATRP, because of the use of thermal initiators as radical source. Reports on the preparation of glycopolymers by NMP are very scarce [10,22].

A variety of surfaces can be used for the preparation of brushes, being the most common ones based on gold, glass, or silicon, which need to be functionalized before the polymerization, for the immobilization of the RDRP initiating sites [22]. Functional gold surfaces to be used in SI-ATRP can be prepared from compounds having two functional groups: (i) thiol, which will be used to anchor the molecule to the gold surface and (ii) hydroxyl, that will allow esterification with the selected ATRP initiator precursor. If the ATRP initiator already possess a thiol functionality, it can be directly attached to the gold surface, thus avoiding the esterification step [24,25]. Glass and silicon substrates can be prepared by the esterification with the acyl bromide or chloride ATRP initiator derivatives or by silanization with ATRP initiators containing a silane reactive group [26,27]. The most chosen initiators are α-bromoisobutyryl bromide or its derivatives, like ethyl α-bromoisobutyrate [28,29]. SI-RAFT is less common for the preparation of glycopolymer brushes. In this technique, the RAFT agent is attached to the surface, which can occur by either the R or the Z groups [22]. As an example, a synthesized RAFT agent was covalently linked to a silicon surface with the OH groups substituted by NH_2_ and further used for the synthesis of well-defined brushes with *N*-acryloyl glucosamine and *N*-isopropylacrylamide (NIPAAm) [30].

Surface modification with thin polymer films forming polymeric brushes is considered one of the most promising ways to obtain “smart” surfaces, which can change some of their physicochemical properties (e.g., solubility) as a response to external stimuli (e.g., pH) or present desired functionality for targeted applications (e.g., carbohydrate-recognition ability by lectins) [17]. On this matter, both SI-ATRP and SI-RAFT polymerization are very robust and versatile techniques to prepare such materials, as they allow the control of different polymer features, like degree of polymerization, brush grafting density, surface geometry, and film thickness at the nanoscale level [17,18,19]. However, the high grafting density can cause steric hindrance problems that are worsen by the brushes restricted mobility imposed by the attachment to surfaces, that in turn can negatively influence the performance of these materials, like their ability to be recognized by lectins [28]. In addition, the characterization of immobilized brushes in terms of molecular weight, is only possible after hydrolysis of the polymer, which is a disadvantage. On this matter, the preparation of bottlebrush polymers could be an alternative to address these limitations. 

Glycopolymer bottlebrushes are usually obtained by “grafting from” of monomers, using a previously prepared multifunctional polymer backbone [31,32,33]. Both backbone and side chains can be synthesized by the combination of RDRP methods, such as RAFT and ATRP [33], or with other polymerization techniques, like ring-opening polymerization (ROP) [32]. For example, a backbone of poly(2-(2-bromoisobutyryloxy) ethyl acrylate) was prepared by RAFT and used as macroinitiator for the polymerization of 6-*O-*methacryloyl-α-d-glucoside by ATRP [33]. The use of RDRP allows the control over the length of the backbone and sides chains, as well as the number and location of initiation sites, thus enabling the precise design of the bottlebrush [20]. The determination of the molecular weight of bottlebrushes is easy, as they are prepared in solution [16,20], contrary to what is observed for brushes immobilized in surfaces. To avoid the cleavage of polymers from the surface, some authors theoretically assume that the control over the polymerization of brushes immobilized in surfaces is similar to the one obtained for a linear polymer synthesized in the same reaction conditions as the brush, but using a sacrificial initiator, which might not correspond to the reality [19]. Bottlebrushes’ tunable topology and functionality make them attractive materials for biomedical applications, such as delivery systems. Nevertheless, more studies to understand the effect of individual structural changes on the performance of the polymers are still required [16]. Unfortunately, to the best of our knowledge, there are no studies comparing the performance of glycopolymer bottlebrushes with glycopolymer brushes immobilized on surfaces. 

## 3. Glucose-Containing Polymer Brushes

**Figure 4:**
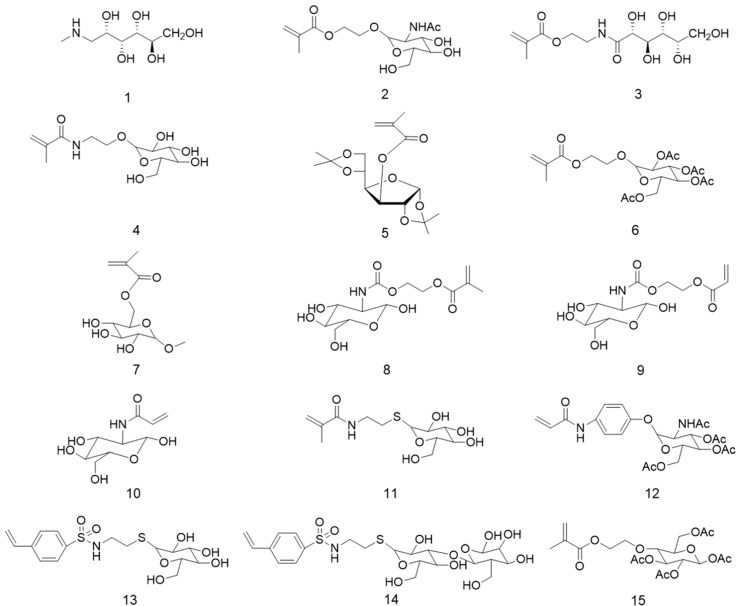
Chemical structure of *N*-methyl-d-glucamine (**1**) and protected and non-protected glucose-based vinyl monomers used for the synthesis of polymer brushes by RDRP. **2**: methacryloyloxyethyl d-glucopyranoside; **3**: d-gluconamidoethyl methacrylate; **4**: 2′-acrylamidoethyl-β-d-glucopyranoside; **5**: 3-*O*-methacryloyl-1,2:5,6-di-*O*-isopropylidene-d-glucofuranose; **6**: 2-(2′,3′,4′,6′,-treta-*O*-acetyl-β-d-glucosyloxy)ethyl methacrylate; **7**: 6-*O*-methacryloyl-α-d-glucoside; **8**: 2-{[(d-glucosamine-2-*N*-yl)carbonyl] oxy}ethyl methacrylate; **9**: 2-{[(d-glucosamine-2-*N*-yl)carbonyl] oxy}ethyl acrylate; **10**: *N*-acryloyl glucosamine; **11**: 2′-acrylamidoethyl-1-thio-β-d-glucopyranoside; **12**: *p*-acrylamidophenyl 3,4,6-tri-*O*-acetyl *N*-acetyl-glucosamine; **13**: 4-vinylbenzenesulfonamidoethyl 1-thio-β-d-glucopyranoside; **14**: 4-vinylbenzenesulfonamidoethyl 1-thio-β-d-lactoside; **15**: 2-(2-,3-,4-,6-tetra-*O*-acetyl-β-d-glucosyloxy) ethyl methacrylate.

Glucose, usually named as dextrose, is a monosaccharide starch-derived sweetener [34] and it is the only energy source for both central nervous system and red blood cells [35,36,37]. The majority of glycopolymer brushes reported the use of glucose derivatives as the saccharide unit, mainly due to glucose bioavailability, biocompatibility, and known recognition ability by different lectins, like Con A, *Griffonia simplicifolia* or wheat germ lectins [5,7,38,39,40]. Most of the glucose-based brushes are obtained by the direct polymerization of the glycomonomer, on its protected or unprotected form, by SI-ATRP from a functionalized surface, using water or alcohol/water mixtures as solvents, CuCl or CuBr as the catalyst and bipyridine as the ligand [17,41,42,43,44]. To the best of our knowledge, there is only one work reporting the preparation of glucosamine-containing brushes by RAFT polymerization from silicon wafer by the Z-group approach [30]. The characterization of the brushes is mainly focused on the determination of grafting density and thickness of polymer layer. Unfortunately, the confirmation of the control over the molecular weight of the glycopolymer is often neglected or assumed to be similar to the one obtained for the homogeneous polymerization of the glycomonomer in a separated ATRP experiment or during the SI-ATRP using sacrificial initiator. In most of the cases, this assumption could be very far from reality. *N*-methyl-d-glucamine and different glucose- and glucosamine-based acrylamide or (meth)acrylate monomers [30,33,43,45,46,47,48,49,50,51], (Figure 4), either in their linear or ring form, have been synthesized and used for the preparation of well-defined polymer brushes by RDRP methods. The resultant materials have been extensively studied mainly for the specific binding and recognition by lectins [22,24,25,38,39,41,48,49,51,52,53,54,55,56,57,58].

**Figure 5:**
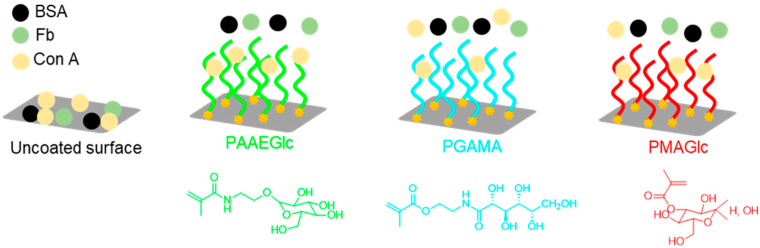
Specific protein interaction of BSA, Fb, and Con A with different glucose-based brushes.

Very hydrophilic poly(2′-acrylamidoethyl-α-d-mannopyranoside) (PAAEM), poly(2′-acrylamidoethyl-β-d-galactopyranoside) (PAAEGal) and poly(2′-acrylamidoethyl-β-d-glucopyranoside) (PAAEGlc) brushes, prepared by SI-ATRP from silicon substrates in water, exhibited a 149-, 172-, and 500-fold reduction of the non-specific adsorption of bovine serum albumin (BSA), respectively, as well as a 52-, 115-, and 135-fold fibrinogen (Fb) adsorption, respectively, in comparison to uncoated silicone surface [38]. Besides the antifouling character of these surfaces, their specific interaction with Con A lectin was evaluated. Results revealed specific interaction between this lectin and both glucose and mannose-containing brushes [38], corroborating results from the literature which show that galactose does not interact specifically with Con A [5,7,8]. A similar behavior has been also observed for two other different glucose-based methacrylate polymer brushes, namely poly(d-gluconamidoethyl methacrylate) (PGAMA) [59] and poly(3-*O*-methacryloyl-d-glucofuranose) (PMAGlc) [60]. To evaluate the influence of the glucose structure on its affinity toward Con A, polymer brushes with glucose in its: (i) linear form (PGAMA), (ii) pyranose form with the introduction of a double bond in the hydroxyl group at the C-3 position (PMAGlc), and (iii) pyranose form with no structural modifications at C-3, C-4, or C-6 positions were studied, (Figure 5) [38]. The authors concluded that the specific binding of Con A and non-adsorption of other proteins are privileged by the presence of glucose in its pyranose form, which is the natural conformation of this sugar instead of the linear form. In contrast, for monomers derived from glucopyranoside, the best results (higher specific adsorption of Con A) were obtained for polymers with no modification of the C-3, C-4, and C-6 hydroxyl groups. These results are in agreement with other reports showing that Con A strongly binds to the hydroxyl groups at C-3, C-4, and C-6 positions of unmodified d*-*gluco- and d*-*mannopyranose [8,38,60]. Taking into account these results, this type of glycopolymeric structures could be promising biomaterials for protein separation processes and modulation of immune system response (e.g., through modulation of the complement system), by specific interaction with lectins [39,41,57,61,62,63,64,65,66]. For example, Kizhakkedathu and co-workers synthesized glycopolymer brushes, based on different carbohydrates, through SI-ATRP from polystyrene (PS) NPs, targeting several grafting densities and accessing their suitability for the modulation of the complement activation via alternative pathway [62]. The authors verified that the complement activation increased for glycopolymer grafting densities above a threshold of 0.031 chains/nm^2^, whereas particle size and polymer concentration did not play a critical role on that process. The results were also influenced by the type of sugar used, with glucose-bearing glycopolymers functionalized NPs demonstrating stronger complement activation than the ones carrying galactose, suggesting that both carbohydrate unit and grafting density are important parameters in the complement activation. This approach can be applied for the design of new drug delivery systems, especially focused in immunotherapies. 

Besides specific interaction with lectins, surfaces modified with glycopolymers can also present other properties, such as antifouling, antimicrobial, or antiviral activity [17,44,49,67,68,69]. Despite the relevance of these applications, the examples reported in the literature using glycopolymer brushes are scarce compared with the ones dealing with linear glycopolymers [9]. This could be an interesting opportunity to develop new materials based on polymers with high surface concentration of active groups, which could potentially exhibit increased performance. Fouling of membranes is common in biomedical applications and, although not fully understood, it is known that this phenomenon is associated with protein adsorption at the surface of the material. For example, a poly(vinylidene difluoride) (PVDF) microporous membrane was modified with PGAMA by aqueous SI-activator generated by electron transfer (AGET) ATRP to introduce higher biocompatibility and antifouling character [44]. Results showed an increase of the hydrophilicity of the membranes after coating with PGAMA, resulting in a non-specific protein adhesion decrease from 0.96 mg/cm^2^ (uncoated PVDF membranes) to 0.19 mg/cm^2^ (PGAMA-coated PVDF membranes) using BSA as model protein [44]. As well the antifouling character of the coating, the biocompatibility of the glycopolymer brushes was confirmed by the one-tenth decrease of the adhesion of platelets to the PGAMA-coated PVDF membranes in comparison to the pristine ones, in fresh human blood test. This type of coated materials could be interesting candidates for components of implant coatings. Because glycopolymers are very hydrophilic, they can present antimicrobial activity because of the formation of a hydration layer, which is known to form a physical and energetic barrier that prevents the adhesion of bacteria to the material (same mechanism that prevents protein adsorption) [70]. By this mean, surface colonization and proliferation of bacteria that could potentially lead to infection could be avoided. Therefore, glycopolymer brushes could be useful to coat medical implants to prevent infection processes after surgery, avoiding the removal of the implant, amputation or sometimes even patient death. This possibility has been demonstrated for glycopolymer brushes prepared by the modification a polyoxyethylene backbone, with different amounts of glycosyl and methyl groups, against different bacteria strains [71]. In addition, the killing effect of *Escherichia coli* (*E. coli*) bacteria on modified gold NPs with poly(2-(methacrylamido)glucopyranose) and poly(2-(methacryloyloxy) ethyl trimethylammonium iodide), synthesized by RAFT, has been reported [72]. The use of RDRP methods for the preparation of glycopolymer brushes, represent a great advantage as the hydration layer could be adjusted by the fine-tuning of the thickness of the brush, thus modulating the antimicrobial performance of the materials. Following this strategy, Vamvakaki and co-workers have prepared films of PGAMA brushes by SI-ATRP from glass substrates. The effect of the brush thickness on cell morphology and cytoskeleton organization and growth was evaluated by culturing MC3T3-E1 pre-osteoblasts on the polymers [17]. Cell growth was observed for films with 4 nm thickness, whereas cell adhesion was prevented by films with thickness higher than 10 nm, due to increased hydrophilicity of the surface. These results confirm the possibility of tailoring the performance of anti-adhesive glycopolymers by using RDRP techniques to control the thickness of the films. 

**Figure 6:**
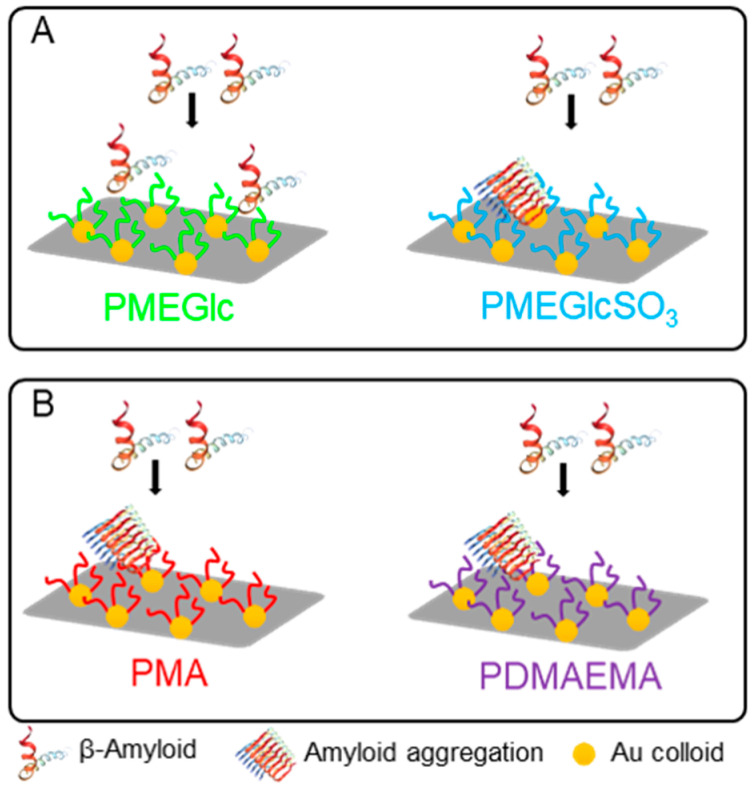
Interaction of β-amyloid with different polymer brushes. (**A**): influence of the presence of the sulfate group on the glycopolymer; (**B**): non-specific interaction of PMA and PDMAEMA by hydrogen bonding and electrostatic interactions, respectively.

In order to further expand the range of applications of glycopolymers, the modification of their chemical structure is sometimes performed to provide specific properties. For instance, sulfonated glycopolymers, in which the OH groups of the sugar are replaced by OSO_3_ groups, have been reported to inhibit the aggregation of amyloid-β peptide (Aβ) [73,74]. Aβ is normally produced by the organism and it is present in the brain, but when its abnormal cleavage occurs, these peptides form aggregates leading to fibrils, which are deposited extracellularly as amyloid plaques. The formation of amyloid plaques is one of the neuropathologic hallmarks associated with the development of Alzheimer’s disease [74]. On this matter, a sulfated poly(2-methacryloyloxyethyl d-glucopyranoside) (PMEGlc) glycopolymer was prepared by normal ATRP in methanol/water mixture near room temperature from a thiolated initiator, followed by grafting of the polymer to a glass substrate functionalized with an Au colloidal monolayer, via disulfide bridge [42]. The sulfonation was performed after polymerization. The grafting of end-thiolated poly(methyl acrylate) (PMA) or poly(2-dimethylaminoethyl methacrylate) (PDMAEMA) homopolymers prepared by RAFT in ethanol was also performed as control materials, aiming to evaluate the effect of either hydrogen bonding or electrostatic interaction, respectively, on the adsorption of Aβ aggregates, (Figure 6). Localized surface plasmon resonance (LSPR) technique and atomic force microscopy (AFM) confirmed the adsorption of Aβ to the sulfated glycopolymer brush, whereas no aggregation was observed for the non-sulfated material. Both PMA and PDMAEMA brushes exhibited non-specific adsorption of Aβ, suggesting that the binding process between Aβ and polymers is influenced not only by specificity, but also by both hydrogen bonding and electrostatic interactions. These preliminary results could be of interest for the future development of diagnose methods for the Alzheimer’s disease based on the use of glycopolymers.

Glucose-containing polymer brushes have been also suggested as candidates for water purification, namely for the removal of boron, which is a micro-nutrient that is important for both plant and animal living activities, but lethal when found in high concentration. World Health Organization guidelines set a level of 0.5 mg/L for irrigation water and 2.4 mg/L for drinking water [75,76,77]. For this, PGAMA was grafted from a chloromethylated polysulfone (CMPSF) membrane through SI ATRP [75]. The authors presented a very complete systematic study, investigating the influence of several parameters, such as grafting density, initial concentration of boron, sorption time, solution pH, and ionic strength on the boron removal percentage and retention efficiency by the membranes. Results showed that the complexation capacity increased with increasing grafting density. The membranes presented the highest retention efficiency for the removal of boron from solution with 300 mg/L or more of this micro-nutrient. For lower concentration of boron (e.g., 5 mg/mL, which mimics sea water), the membranes were less effective (only 5% of retention efficiency). Nevertheless, the authors claimed that, under optimized conditions, the membranes showed superior performance than most commercially available resins for this application [75]. This work clearly demonstrates the potential of RDRP methods for the preparation of polymers with tailor-made performance.

## 4. Mannose-Containing Polymer Brushes

Mannose is a naturally occurring aldohexose required in the human organism for protein glycosylation, and it is used for the therapy of congenital disorder of glycosylation, as well as in the prevention of urinary tract infections caused by *E*. *coli* [78,79,80,81]. There are innumerous mannose-based receptors found in cells involved in inflammation and immune events, like dendritic and endothelial cells [82].

**Figure 7:**
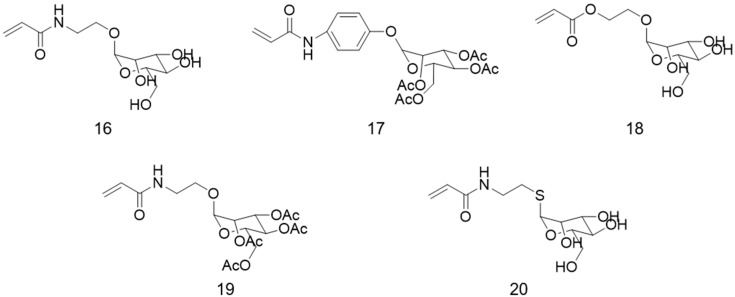
Structures of protected and non-protected mannose-based vinyl monomers used for the synthesis of polymer brushes by RDRP. **16:** 2′-acrylamidoethyl-α-d-mannopyranoside; **17:** p-acrylamidophenyl 2,3,4,6-tretra-*O*-acetyl-α-d-mannopyroside; **18:** 2-methacryloyloxyethyl d-mannopyroside; **19:** 2′-acrylamidoethyl 2,3,4,6-tetra-*O*-acetyl-α-d-mannopyranoside; **20**: 2′-acrylamidoethyl-1-thio-(2)-d-mannopyranoside.

Polymer brushes containing mannose-residues, (Figure 7), are also found in the literature and have been mostly applied for lectin recognition, including interaction studies [32,83,84,85,86,87,88]. A quantitative analysis of the influence of the spatial distribution of carbohydrates on copolymer brushes with mannose derivative (2′-acrylamidoethyl-α-d-3-mannopyranoside) and galactose derivative (2′-acrylamidoethyl-β-d-galactopyranoside), and the respective homopolymers (control samples), on the binding kinetics with Con A was reported [83]. The glycopolymer brushes were prepared by SI-ATRP from Au chips with grafting density, carbohydrate composition, and the degree of polymerization precisely controlled. No affinity was found between galactopyranoside and the lectin, which is in agreement with other literature reports [5,8,25,87]. On the other hand, glycopolymer brushes with mannopyranoside presented specific multivalent interaction with Con A. For the copolymers, the association constant (*K*_A_) for the binding with Con A increased from (3.8 ± 0.6) × 10^3^ M^−1^ to (1.2 ± 0.5) × 10^5^ M^−1^, with the increase of the mannose content from 0.8% to 9%, showing the great influence of the concentration of this sugar on the binding process. However, all the copolymers presented weaker interaction with Conv A than the one observed for the mannopyranoside-homopolymer, with *K*_A_ of (3.1 ± 0.5) × 10^6^ M^−1^, for a grafting density of 0.10 chain/nm^2^. The *K*_A_ of the copolymer with 0.8% of mannose was similar to the one binding between free methyl α-d-mannppyroside and Con A, being the mechanism based on a monovalent interaction [83].

**Figure 8:**
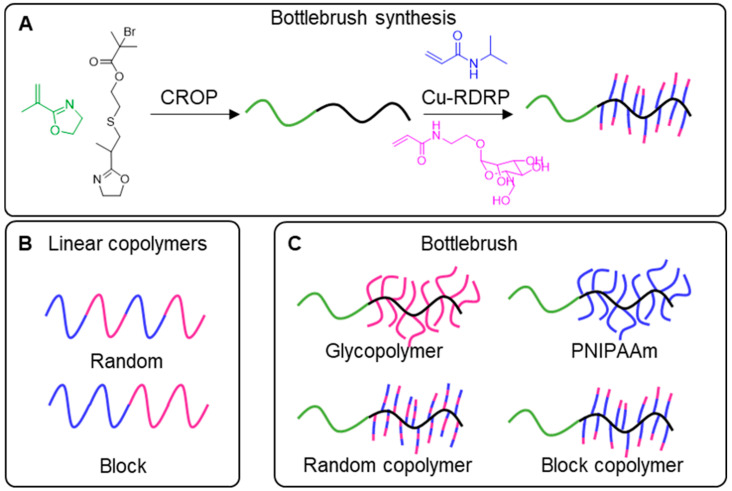
Schematic representation of the synthesis of oxazoline-based and mannose-containing linear and bottlebrushes copolymers. (**A**): Synthesis of copolymer bottlebrushes by combination of CROP with RDRP techniques; (**B**): representation of random and block linear copolymers; (**C**): representation of the different bottlebrushes with side chains of vary compositions, namely homopolymers (glycopolymer and PNIPAAm), random and block copolymers [89].

Amphiphilic brush-like copolymers containing mannose have been prepared through the copolymerization of poly(ethylene glycol) (PEG)- and poly(ε-caprolactone) (PCL)-based macromonomers by ATRP, followed by functionalization of the PCL side branches with mannose derivative through a Cu(I)-catalyzed azide-alkyne cycloaddition (CuAAC) click reaction [32]. Turbidity tests for the incubation Con A with mannose, mannose-free polymer, linear glycopolymers (mannose valency 1 or 2), and branched glycopolymer (mannose valency 4 or 8), showed that the binding only occurred for glycopolymers with mannose valency higher than 1. Precipitate of glycopolymer-Con A aggregates occurred faster for the linear glycopolymer with divalent mannose, with initial rates of Con A precipitation of 0.75 (AU/min). Regarding the branched structures, with mannose valency of 4 or 8, higher mannose content represented a lower bonding affinity with Con A, with initial rates of Con A precipitation of 0.0.41 and 0.32 (AU/min), respectively. The authors showed that the binding between the polymers and Con A could be adjusted by manipulating both the macromolecular architecture (linear or star-shaped) and the mannose valency (1, 2, 4 or 8). These are very interesting glycopolymer brushes which are not attached to surfaces, as it is common to find in literature reports. These structures can self-assemble in water having the potential to be used as drug carriers for cell-targeted delivery. Very recently, Beyer and co-workers developed bottlebrush copolymers with affinity for two lectins associated with immunologic response, namely mannose-binding lectin (MBL) and dendritic cell-specific intercellular adhesion molecule-3-grabbing non-integrin (DC-SIGN) [89]. The authors synthesized a series of random and block bottlebrush copolymers through the combination of cationic ring-opening polymerization (CROP) of 2-isopropenyl-2-oxazoline (backbone) and grafting from of NIPAAm and 2′-acrylamidoethyl-α-d-mannopyranoside (ManAA) (side chains) by aqueous Cu-mediated RDRP (Figure 8). Control samples consisting of linear PNIPAAm-*b*(*r*)-PManAA copolymers were also prepared by Cu-mediated RDRP. The lectin binding studies showed that brush copolymers had higher *K*_a_, in comparison with the linear ones, which was attributed to the higher sugar density (Table 1). Regarding the bottlebrush architecture, higher affinity was observed for homopolymer side chains with longer glycopolymer segments. For the brushes with side chains composed of copolymers, higher affinity was achieved when the PNIPAAm segments were closer to the backbone, not shielding the glycopolymer block. MBL and DC-SIGN protein studies proved that these complex architectures have higher affinity toward lectins and that the arrangement of the polymer segments is important to improve the binding between glycopolymers and lectins. All copolymers were also tested for the encapsulation of an inexpensive hydrophobic drug mimic, 1,4 dihydroxyanthraquinone (DHA), at 25 °C and 37 °C. In general, linear copolymers presented lower amount of encapsulated DHA, and drug loading increased with the increasing of temperature for all types of copolymers. Regarding the bottlebrush block copolymers, those containing temperature-responsive PNIPAAm at the periphery (far from the backbone) were not able to encapsulate DHA, contrary to the analogues having PManAA segments at the periphery. These results show the importance of both polymeric architecture and spatial arrangement of the polymeric segments on the performance of these drug carrier candidates with affinity toward lectins.

**Figure 9:**
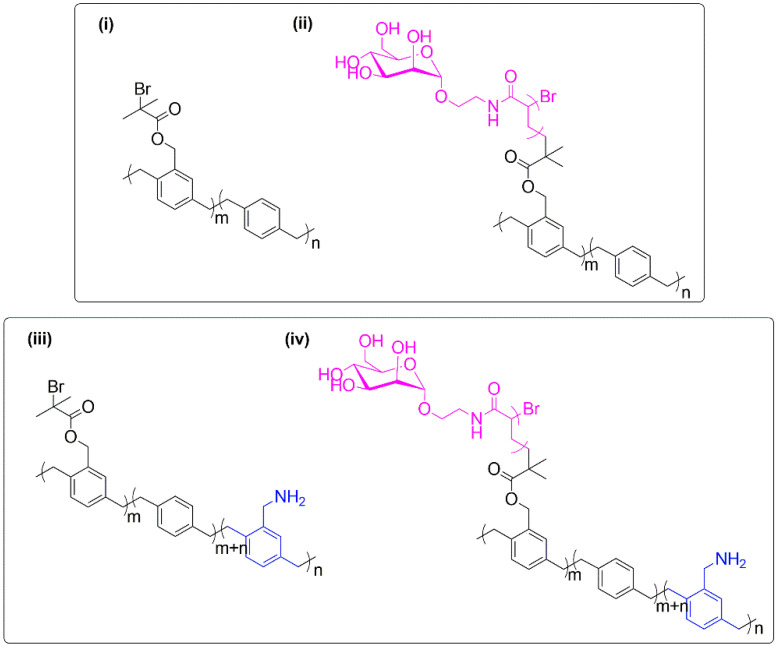
Structures of antiviral glycopolymer brushes prepared by combination chemical vapor deposition polymerization and SI-ATRP: (i) and (ii) reference polymer and correspondent mannose-based glycopolymer brush, respectively; (iii) and (iv) reference copolymer and correspondent mannose-based glycopolymer brush, respectively [18].

Preliminary studies also releveled that mannose can interact with the FimH (mannose specific bacterial lectin) receptors, thus preventing or diminishing the adhesion of bacteria on mannose-containing surfaces [86]. In this vein, mannose-based polymer brushes have been also proposed for the preparation of antiviral surfaces [18,69]. Chemical vapor deposition polymerization was used to synthesize bromine-functionalized coatings, from which mannose-based glycopolymer brushes were grafted by SI-ATRP [18]. Aminomethyl (AM) groups were also introduced in the coating to evaluate the influence of the heterogeneity of the surface on its performance, mimicking the positively charged regions that sometimes are created during the synthesis of glycopolymer brushes. It is important to understand the contribution of the binding between these free cationic groups and the negative charged bacteria, viruses and some proteins, at physiological pH. For the systematic study, four samples were prepared (Figure 9): (i) reference polymer; (ii) mannose-based glycopolymer brush; (iii) reference copolymer; and (iv) mannose-based glycopolymer brush. Among other characteristics as non-specific adsorption of Fb, influenza H1N1 and adenoviruses was evaluated by quartz crystal microbalance measurements.

Results showed that coatings containing only AM groups promoted viral adhesion (Figure 9, (iii)), which could be suppressed by the addition of thick and dense glycopolymer brushes to the coating (Figure 9, (iv)), forming a steric and hydration repulsion barrier. Control polymer brushes containing either glucose or galactose exhibited similar results for the adsorption of all biological molecules tested, suggesting that the non-specific adsorption is not dependent on the stereochemistry of the carbohydrate unit present in the brushes. 

## 5. Galactose-Containing Polymer Brushes

Galactose is a monosaccharide obtained from lactose, with chemical structure that resembles glucose. After being produced in the organism by the enzymatic breakdown of lactose, galactose can be transformed in glucose, by enzymatic reactions, and enter the glycolysis pathway, thus becoming an energy source [90,91].

**Figure 10:**
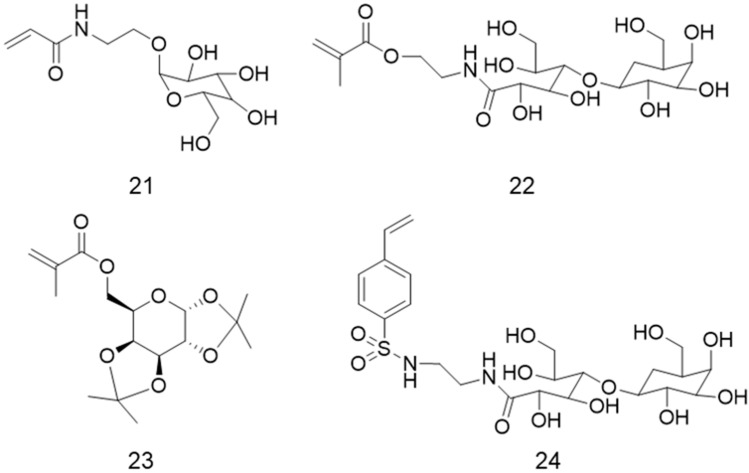
Chemical structure of galactose-based vinyl monomers used for the synthesis of polymer brushes by RDRP. **21:** 2′-acrylamidoethyl-6β-d-galactopyranoside; **22:** 2-lactobionamidoethyl methacrylate; **23:** 6-*O*-methacryloyl-1,2:3,4-di-*O*-isopropylidene-d-galactopyranose; **24**: *N*-[2-(4-vinylbenzenesulfoneamido)ethyl] lactobioneamide.

Although galactose has no specific interaction with Con A lectin, it can recognize specific domains of both *Ricinus communis* (RCA_120_) and peanut agglutinin (PNA) lectins [38,83,88,92,93,94,95]. RCA_120_ is purified from castor seed plants and it is widely used for the study of glycoproteins on the cell surface and for the separation of glycoproteins by affinity chromatography [93,95]. PNA is a lectin that specifically labels photoreceptors in different vertebrate retina [96]. This structure can also interact with human peripheral blood lymphocytes [97], which makes it suitable for retinal drug delivery or as a marker for red blood cells. Some galactose-based vinyl monomers (Figure 10) have been synthesized to further prepare galactose-based polymer brushes by RDRP techniques, which have been evaluated for different applications, namely protein separation.

Glycopolymers with galactose, mannose, and glucose were prepared by combination of ATRP and CuAAC click reaction, followed by their grafting to gold NPs. A systematic study using Con A (lectin for the specific recognition of glucose and mannose), PNA (lectin for the specific recognition of galactose), and BSA (protein for the non-specific recognition of carbohydrates units), revealed that galactose-containing polymer brushes exhibited specific interaction with PNA, while both glucose and mannose glycopolymer brushes, as expected, presented specific interaction with Con A [94]. Regardless the sugar moieties, the glycopolymer brushes resisted to BSA adsorption, which is explained by the hydration layer generated by the polymeric chains, successfully blocking the adsorption of proteins without affinity binding domains. The potential of galactose-containing brushes as biosensors for PNA separation and quantification was proved [94]. 

**Figure 11:**
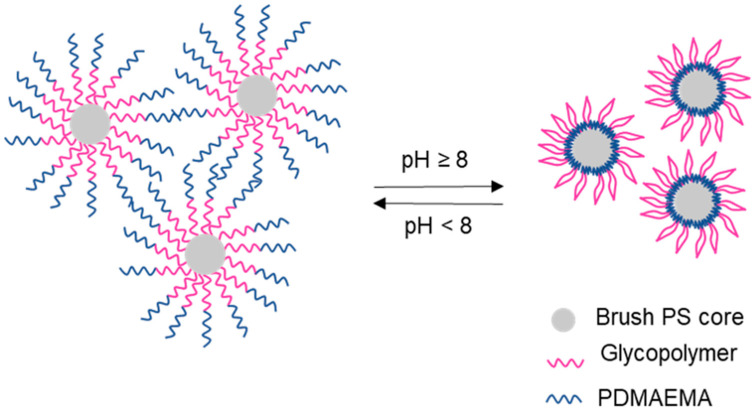
pH-responsive behavior of PMAGal-*b*-PDMAEMA block copolymer brushes at T > LCST.

Another common application for galactose-containing polymers is tumor-targeted therapy [27,31,98,99] because of the ability of galactose to recognize and target hepatocellular carcinoma cells (HepG2) [31,100,101]. As an example, a fluorescent galactose-containing glyco(co)polymer brush was synthesized to improve tumor-targeted photodynamic therapy, which uses photosensitizers and light to generate reactive oxygen species (ROS) that can induce cell death [31]. First, a photosensitizer metal complex Ir(III)-terminated bromine multifunctional poly(benzene-*alt-*fluorene) ATRP macroinitiator (PFF-Ir) was used to block the copolymerization of oligo(ethylene glycol) methacrylate (OEGMA) and glycidyl methacrylate (GMA) by ATRP (PPF-Ir-*g*-(POEGMA-*b*-PGMA)). The final glycopolymer brushes were obtained by functionalization of the PGMA segment with galactose units by CuAAC click reaction (PPF-Ir-*g*-(POEGMA-*b*-PGal)). In vivo assays in mice showed the inhibition of xenograft tumors, confirming the feasibility of the developed glycopolymer brush to induce cellular death in HepG2 cells [31]. A poly(6-*O*-methacryloyl-1,2:3,4-di-*O*-isopropylidene-d-galactopyranose)-*b*-PDMAEMA block copolymer (PMAIGla-*b*-PDMAEMA) was grafted by ATRP from functional PS NPs to form smart spherical glycopolymer brushes as carrier candidates for the targeted drug delivery into the liver [99]. The galactose units (MAIGla) were used to target liver cells, while the cationic PDMAEMA segment, which is both temperature- and pH-sensitive, was chosen to induce damages through electrostatic interaction with the anionic surface of the cancer cells. Dynamic light scattering and cryogenic transmission electron microscopy proved that at pH = 8, and above the critical solution temperature (LCST) (40 °C) of the polymer, there was a decrease on the hydrodynamic radius of the polymer, because of the collapse of the PDMAEMA block (p*K*_a_ ≈ 6) into the brush core, exposing the glycopolymer block to the surrounding environment (Figure 11) [99,102]. Supported by this behavior, the authors postulated that if the synthesized brush entered the blood stream, which has pH = 7.4, the PDMAEMA block would collapse, exposing the galactose-containing inner block and turning the polymer into an efficient targeting agent of liver cells. After contacting with the tumor, which has pH = 5, the cationic block (PDMAEMA) would expand, damaging the anionic surface of the tumor [99]. Unfortunately, the authors did not conduct either in vitro or in vivo tests to confirm this hypothesis. 

Lactobionic acid (LBA) is a disaccharide composed of galactose and gluconic acid, which has been widely polymerized, in the form of vinyl monomer, to give materials for biomedical applications, mainly because of its ability to target HepG2 cells [24,103]. On this matter, copolymer brushes containing both LBA and NIPAAm exhibited specific adsorption of RCA_120_ (*K*_A_ of 1.86 × 10^5^ M^−1^ for PLAMA brushes) [53] and adhesion of HepG2 cells at 37 °C [27]. Because of the thermo-responsive character of PNIPAAm, for temperatures above the polymer LCST (32 °C), these polymeric segments turned hydrophobic and collapsed, allowing the interaction between the exposed glycopolymer block and HepG2 cells. For temperatures below the LCST, PNIPAAm block is hydrated and the polymeric chains extended resulting in the detachment of the HepG2 cells. This approach can be used for the design of “on-off” cell capture/release substrates for biomedical applications, such as cancer diagnostic and regenerative medicine [27]. Although LBA-based polymeric structures have been mainly explored for the recognition of HepG2 cells, some studies have also investigated their affinity toward galactose-recognition lectin RCA_120_ [26,27,28,53,98,104,105,106,107]. For example, Au chips were modified with ATRP initiators for the direct polymerization of 2-lactobionamidoethyl methacrylate (LAMA) by SI-ATRP. The influence of the grafting density, epitope density, and thickness of the polymer brushes on their affinity toward RCA_120_ was evaluated [28]. Results showed that the interaction between the polymers and RCA_120_ did not improve with the increase of the brush thickness for values above 10 nm, because of the high steric hindrance which blocked the access of the protein to the carbohydrate residues. The influence of the grafting density was evaluated by varying the percentage of surface modification with polymer brushes having similar thickness. Results showed that there was an increase of the interaction between polymer and lectin, as the surface modification increased up to 40%, due to the increase of the galactose content of the surface. Above that value, the interaction between polymer and RCA_120_ was prevented also due to the increase of steric hindrance. For the epitope sugar density tests, random copolymers of LBA with a biocompatible monomer, namely 2-hydroxylethyl methacrylate (HEMA), were prepared. RCA_120_ adsorption increased with increasing epitope of LAMA, for contents of LAMA in the copolymer below 40%. Although it is expected the increasing of lectin adsorption as higher percentages of carbohydrates residues are used [28,108], in sterically hindered systems (e.g., glycodendrimers or glycopolymer brushes grafted on surfaces), the limited protein mobility through the brushes and steric hindrance will prevent the interaction between glycopolymer brushes and lectins.

**Figure 12:**
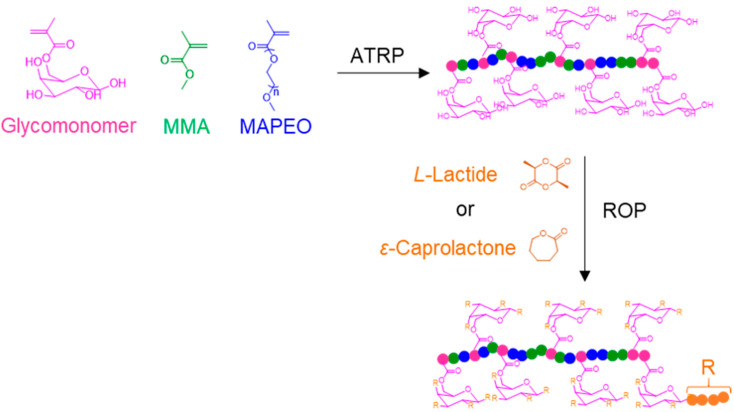
Preparation of a glycol(co)polymer brush by the combination of ATRP and ROP polymerization techniques.

There is one example of galactose-containing glycopolymer brush-like structure, synthesized by the combination of ATRP and ROP, that is not attached to a surface [109]. For this, a random glyco(co)polymer containing methyl methacrylate (MMA), α-methoxy, ω-methacrylate (ethylene oxide), and a methacryloyl-d-galactopyranose-based monomer was prepared by ATRP. Next, a brush-like structure was obtained by performing ROP of ε-caprolactone or *L*-lactide from the free hydroxyl groups of the galactose residues (Figure 12). The authors performed a complete characterization of the glycopolymer structure by nuclear magnetic resonance, size exclusion chromatography, and Fourier-transform infrared spectroscopy. Unfortunately, no application was suggested by the authors for this new brush-like polymer. In addition, it would have been interesting to evaluate the influence of the steric hindrance of this type of “less blocked” structure, in comparison to the ones attached to surfaces, on the binding of lectins.

PLAMA-based polymers could be also used for the preparation of sensors. As an example, a gold colloid-based LSPR sensor was constructed by grafting of either PLAMA or a monovalent galactose ligand-based vinyl monomer, by ATRP, and their interaction with RCA_120_ was studied [98]. Both polymer brushes adsorbed RCA_120_, but the absorbance registered for the PLAMA brushes was higher than the one observed for the brushes with galactose. This outcome can be explained by the multivalent ligand character of PLAMA, which can bind to the lectin in a three-dimensional way, with *K*_A_ of 8.5 × 10^6^ M^−1^ for DP = 32.8. The reversible adsorption of the lectin was investigated by immerging the sensors in lectin and lactose solutions alternatively, to access the reusability of these polymer brushes. Unlike the brush with monovalent galactose, the PLAMA brush showed non-specific adsorption and reversible adsorption of RCA_120_, suggesting that the PLAMA brush-based LSPR sensor could be reused. 

The use of glycopolymers to increase hydrophilicity of polymers is an interesting way to expand the range of application of such materials, namely in the biomedical field. Poly(ethylene terephthalate) (PET) is one of the most common thermoplastic polymer, which is used in different areas, such as textile industry and packing, and whose applications are limited by its hydrophobic character. Lepoitteven and co-workers have evaluated the improvement of PET hydrophilicity by the SI-ATRP grafting of LAMA, forming brush-like structures [29]. The surfaces were characterized by water contact angle and the authors concluded that the PET surface wettability was dependent on the type of functionalization and grafting density. By manipulating the monomer/initiator ratio, it was possible to obtain surfaces with different properties ranging from hydrophilic to superhydrophilic, with no deleterious effect on the PET bulk properties.

## 6. Other Carbohydrate-Based Polymer Brushes

**Figure 13:**
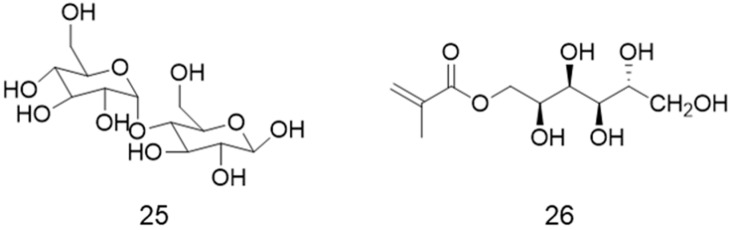
Chemical structure of maltose (**25**) and sorbitol methacrylate (**26**) used for the synthesis of polymer brushes by RDRP.

As the most studied lectins are the ones with specific recognition of glucose, mannose, and galactose, other sugars have not been commonly applied for the preparation of complex glycopolymeric structures. To the best of our knowledge, there are only two examples of maltose- or sorbitol-containing polymer brushes reported in the literature (Figure 13) [110,111].

Maltose is a disaccharide composed of two units of glucose joined by a glyosidic linkage, found in germinating grains and acts as an intermediate for the intestinal digestion of glycogen [112,113]. Gold substrates were modified with an ATRP initiator for the preparation of a POEGMA brush by SI-ATRP, followed by the functionalization of the polymer with maltose [110]. The saccharide was further modified by enzyme-catalyzed elongation, turning the branch ramification of the polymer brush in dextran. Brushes of POEGMA, maltose-POEGMA, and dextran-POEGMA were then tested for the recognition by BSA, RCA_120_, and Con A. The sugar-free POEGMA brush did not interact with any of the investigated proteins (adsorption lower than 3.0 ng/cm^2^), confirming its high resistant character for protein adsorption. The same behavior was exhibited by the sugar-containing polymer brushes for the absorption of both BSA and RCA_120_. On the other hand, high absorption values were obtained for the interaction with these glycopolymers with Con A (1918.5 ng/cm^2^ for maltose and 2826.8 ng/cm^2^ for dextran). The higher recognition ability by the dextran-POEGMA brush (*K*_A_ 3.99 × 10^4^ M^−1^) was attributed to the increased density of glycopolymeric chains and their proximity with the protein recognition domains. Both maltose and dextran-based polymer brushes could be interesting candidates for application in protein separation processes and modulation of immune system response. 

Sorbitol is a sugar-derived alcohol commonly used as an excipient in drug formulations and as a sugar substitute to sweeten candies, gums, and baked goods [114]. Potential antiviral sorbitol-containing polymer brushes grafted from surfaces have been prepared by the same research group that reported the synthesis of mannose-based brushes for antiviral purposes (Figure 8) [18], following a similar strategy (i.e., comparing the performance of the sugar-based brush in the presence and absence of AM groups, which can act as binding sites for virus) [111]. The authors started this study by testing the sorbitol-free surface and found that increased concentration of AM groups led to faster adsorption of virus-like NPs. With the addition of the glycopolymer brushes, the adsorption rate decreased because of the lower availability of AM binding sites. Unfortunately, the investigation about the influence of the grafting density and thickness of the sorbitol-based polymer brushes was not performed. It would be also interesting to compare the performance of both sorbitol- and mannose-containing brushes for this application.

## 7. Conclusions and Outlook

Carbohydrates are biocompatible and naturally occurring molecules, capable of being recognized by specific lectins, which are involved in different biological processes. The most studied lectins are Con A, PNA, and RCA_120_, each one having different and specific sugar recognition domains. While Con A specifically recognizes both glucose and mannose units and for that reason it is the focus of most of the works reported, PNA and RCA_120_ are able of specifically interact with both galactose and carbohydrates with a galactose unit, namely, LBA. The premise for higher success in protein recognition of carbohydrates is to use them in the pyranose form, with free hydroxyl groups at C-3, C-4, and C-6 positions. The interaction between lectins and carbohydrates is enhanced by the binding of the multiple carbohydrate units to the protein, also known as the multivalent effect. In addition, this interaction can be also augmented by using complex and well-defined glycopolymeric structures (e.g., brush-like), in comparison to linear analogues or simple free sugar units.

Despite the control over several variables, focus is mainly given to understanding the effects of manipulation the grafting density and thickness of the glycopolymers brushes on their performance, parameters such as molecular weight distribution and distribution of the polymeric chains on the surface are often neglected. 

The majority of the glycopolymer brushes reported in the literature have been synthesized by SI-ATRP, which allows a stringent control over the brush molecular weight, molecular weight distribution, architecture, degree of polymerization, surface geometry, thickness and grafting density. Despite the possibility to control all these parameters, focus is mainly given in understanding the influence of the grafting thickness and density on the glycopolymer brush performance. Generally, the increase of both grafting thickness and density have shown to improve the affinity of glycopolymer brushes with specific carbohydrate recognition domains in lectins. However, because of the limited mobility of the chains, inevitable steric hindrance problems arise in surfaces with high grafting thickness and density, thus decreasing the potential of the material. Future research on this topic should be focused on the development of polymerization systems suitable to synthesize brushes in the bottlebrush form, using unprotected glycomonomers and aiming to understand how the brushes structural properties influence their recognition by lectins. Specifically, more studies providing information about the influence of some polymer properties, that are often neglected (e.g., molecular weight distribution), on the performance of the polymers are highly desirable. These studies would facilitate future design of glycopolymers, which could potentially exhibit improved specific recognition by lectins and be used as drug/gene carriers or even polymers with intrinsic therapeutic activity.

Glucose-containing polymer brushes are extensively studied for the specific interaction with Con A, making them suitable for protein separation. Other applications such as antiviral or antibacterial surfaces or diagnostic markers for Alzheimer´s disease have also been explored. Mannose is the most complementary sugar for Con A carbohydrate recognition site and most of the polymer brushes-bearing mannose are used for protein separation. These materials can selectively adsorb Con A, in detriment of other proteins and can also successively block virus and bacterial adherence to surfaces. Polymeric structures containing galactose do not recognize Con A, but they can recognize other proteins, such as RCA_120_ or PNA, making them good candidates for protein detection and separation, based on these specific interactions. Because of the ability of this carbohydrate to recognize HepG2 cells, galactose and LBA-containing brushes have been also investigated for diagnostic and tumor-targeted therapy. Despite the number of applications pointed out for glycopolymers, there are not many works exploring the potential of brush-like architectures. It is then of great interest to deepen the knowledge regarding the mechanism of action of these polymers and to synthesize tailor-made structures to achieve their full potential.

## Figures and Tables

**Table 1:** Association constants (*K*_a_) between MBL and DC-SIGN lectins and PNIPAAm- and PManAA-based (co)polymers with different architecture and composition.

Architecture	Polymer	*K*_a_ (×10^−3^ Ms^−1^)	
		MBL	DC-SIGN
Linear	PNIPAAm_10_-*b*-PManAA_10_	0.575	1.53
	PNIPAAm_50_-*b*-PManAA_10_	0.478	1.61
	PNIPAAm_10_-*r*-PManAA_10_	0.364	1.31
	PNIPAAm_50_-*b*-PManAA_10_	0.468	1.58
Homopolymer brush	PNIPAAm_10_	-	-
	PNIPAAm_50_	-	-
	PManAA_10_	1.72	4.66
	PManAA_50_	5.20	27.3
Random copolymer brush	PNIPAAm_10_-*r*-PManAA_10_	1.30	5.52
	PNIPAAm_50_-*r*-PManAA_10_	1.21	3.38
Block copolymer brush	PNIPAAm_10_-*b*-PManAA_1_	0.48	1.73
	PNIPAAm_10_-*b*-PManAA_10_	1.66	4.75
	PNIPAAm_50_-*b*-PManAA_1_	0.43	1.62
	PNIPAAm_50_-*b*-PManAA_10_	2.12	3.69
	PManAA_10_-*b*- PNIPAAm_10_	1.61	4.16
	PManAA_10_-*b*- PNIPAAm_50_	1.19	3.59
